# Trials and Tribulations of Dentists Fifty Years Ago

**Published:** 1908-07-15

**Authors:** Charles H. Bartlett


### THE TRIALS AND TRIBULATIONS OF DENTISTS FIFTY YEARS AGO.

BY DR. CHARLES H. BARTLETT.

Paper read before the West Virginia Dental Society at Parkersburg, W. Va., October 9, 1907.

GENTLEMEN AND FELLOW SUFFERERS:

I was pleased with the compliment of being asked to read a paper before this distinguished and honorable body of my professional brothers. At the same time I fully realize my inability to contribute as much to your pleasure and entertainment, as many others here present might do. However, I could not well refuse the call, without being set down as a shirker, and some one might say "Every body works but father." So if you are tired and weary before the end, please charge it up to the mistaken judgment of some one or two of the Executive Committee.

In selecting a theme, I was somewhat at a loss; fully realizing the fact that I could say nothing new or startling, about Theory and Practice, Treatment of Abscesses and favorable results; Anesthesia of the pulp by pressure, extraction and immediate fillings of root canals; preparation of cavities and correct way of filling; the best kind of filling material; preparation of cavities for porcelain or gold inlays; how to insert them so they will stay, and be better than the old time way of doing things, and almost every thing coming into the daily life of a busy practitioner. All these, and many others things have been so much written about, that I could not think of imposing upon your good nature with a repetition.

I finally settled upon a narrative a little out of the usual order of papers read before the Dental Societies; "The Trials and Tribulations of a Young Dentist of Fifty Years Ago."

I will endeavor to give you the actual experience of a young friend with whom I was very intimate, during this early life, and knew a great deal about his failures and successes, and the true inwardness of his desperate struggle for existance.

Starting in dentistry in 1857 was quite a different thing from starting in 1907.

This paper is more especially intended for the young men, that they may better understand and appreciate the advantages they have at the present day, over the young man of fifty years ago Such reminiscences may not particularly interest such back numbers as Dr. Harrison and Dr. Dowden, and possibly some of our friends from Pittsburg.

This young man was not like many of the young men now entering the profession, with the brilliant hope and expectation of making a half-million the first five years. He started with the modest hope and expectation, of making a living and paying his debts in the course of five or six years. He had nothing to lose but everything to make.

When the boy was fourteen years old his father failed in the mercantile business. He remained at home until he was sixteen, attending school, working night and morning in a dry goods store.

He then went to live with a relative, who was a Physician and Dentist. In thoses days in small towns, both professions were practiced by the same person. It was not as much of a specialty then as at the present time.

He commenced the study of anatomy, physiology and such other branches as would fit him for entering a medical college. His ambition was to be a doctor. At the same time he was pursuing his studies, he was attending school; being old enough to realize the importance of improving every opportunity within his grasp. He was a busy boy. At nineteen he was sent to a reputable academy to finish his limited education. Having devoted more time to the study and practice of dentistry than to the study of medicine, he started at twenty years of age in the practice of dentistry, with the hope and expectation of some day completing a medical course, with dentistry to help him through. Alas, he never reached the goal.

In the winter of 1856 he located in a small town in the northwestern part of New York. At that time he had not had the advantages of a college course that you young men have had, nor did he have the money to start in business. He had few instruments most of them of his own make. He learned to point and temper all his own excavators and pluggers.

He found in this little city of four or five thousand people only one dentist. He wanted to sell out to go farther west. His price was $150.00 (some of you young men may say that wasn't much)—well, there wasn't much to buy, however, $150.00 at that time was about equal to $1,500.00 at the present day. It seemed to him a good big pile of money.

He found a friend in a successful young physician who loaned him the desired amount. He then began to feel his importance as a professional man established in business, as successor to a popular dentist, with, as he thought, flattering prospects.

His office furniture and fixtures were very limited; a two-ply carpet on the floor, four common wood bottom chairs, one cane seated rocker, a small table and a high-backed upholstered rocker used for an operating chair. Did not have the Wilkerson New Model, the Columbia, Harvard, Archer or any other of the many chairs on the market at the present day to select from. His operating outfit was small, few excavators, hand burs and pluggers. In his small laboratory was a charcoal furnace for melting gold and silver, a rolling mill for rolling out gold and silver plate. Very few laboratory tools and appliances. All prosthetic work was done in gold or silver at that time. I dare say there are some here who never saw a hand bur and do not know what it is. The bur was the same we now use with our engine. It was attached to, or rather it was made on the point of an excavator handle, and rotated between the fore finger and thumb, resting one end in a small cup in the hollow of the hand, attached to a ring worn on the middle finger. Rather a slow process for burring out a cavity. An engine would more thoroughly do in three minutes what could be done in half an hour to an hour with the hand-bur and excavators.

No rubber dam to keep cavities dry, he had to use napkins, cotton and bibulous paper. No electric, automatic or engine mallet, putting in all fillings by hand pressure. Often have I seen blisters on his forefinger and thumb, caused by the friction of the hand plugger while inserting and condensing a gold or tin filling. In those days tin was much more used than at the present. He used to make his amalgam fillings from a silver half dollar, filing off with a coarse file what he would use for each filling.

After spending one year in this little town, and not making money enough to pay his board and office rent, he concluded he had better follow the example of his illustrious predecessor, go west and grow up with the country. This was his first failure.

The foundation of success is failure. If there were no failures there would be no successes.

To get out of town he had to increase his indebtedness by borrowing more money. He was able to make a small loan from a friend and started for the wild west. His objective point was Davenport, Iowa. At that time it was as far west as San Francisco is to-day. We had no Southern, Central or Northern Pacific Railways to bring the Western Coast so near the East as it now is.

His loan was too small and he found himfelf stranded at Coldwater, Michigan, the first week in January, 1857. Providence had so arranged that when he stepped off the train, he met an old friend who was keeping a hotel. He took the young man in. The next morning he started out for work, at first he did not succeed, but by persistent importunity, finally succeeded in getting a trial job with Dr. Baker who was running a Daguerreotype and Dental business together. The work was a full upper and lower denture on gold that had not been satisfactory. If successful, he was to receive $12.00. If not his time and labor were lost. He was to take the old gold plates, melt over the gold, role it out, swage the plates and do everything from beginning to finish. I remember hearing him tell how earnestly he prayed, and how faithfully he worked, the whole week, and how, when he took the finished work to the lady, he went with fear and trembling. The Lord answerd his prayers, the fit was good, the lady was pleased, and the young man got his $12.00.

That was a start in the right direction. This brought him more work as the Doctor had several cases of the same kind, and so for four weeks he was kept busy at $10.00 a week, board and washing.

This gave the young man fresh courage. Part of this money he spent to replenish his wardrobe and started on his way rejoicing.

The last of February, 1857, about 7 o'clock one evening he, arrived at the City of Rock Island on the Mississippi River, directly opposite Davenport, Iowa. With his last seventy cents in his pocket he went to a second-class hotel for the night. He started up to his room without supper, with the porter and a tallow dip to show him the way,. He noticed a tray on the floor, by the door of some guest who had had their supper sent to their room. On the tray were three biscuits. These he appropriated without notice of the porter, so there was no arrest for stealing. In after years I have heard him tell the story, of how sweet the little dry biscuits were, and how satisfactorily they partially filled the aching void.

The next morning he crossed the Mississippi River on the ice. In Davenport he found a friend from his New York town who kindly loaned him $5.00, with which he returned to the hotel and paid his bill. Davenport was then a town of about 14,000, with four dental offices. He canvassed the city for work. At first not meeting with much encouragement, but finally he secured another trial job in the laboratory. The Lord blessed his effort, and for six months he maintained himself on laboratory work from two of the principle offices.

Then he concluded to open an office on his own account, and sent for his office furniture and fixtures. He made a very satisfactory beginning, but was too young and inexperienced to know a good thing when he had it. He was just making ends meet, paying his board and office rent, but had not been able to pay anything on his borrowed capital. Feeling somewhat discouraged he concluded to make another move. This was a mistake—he should have stuck to his bush, his business was growing with good prospects for a lucrative practice. Davenport has at this time about 40,000 population.

This next and third move proved a failure. He made one more move. By this time he had learned that "a rolling stone gathers no moss."

Although this was not the place of his choice, being a small town of only about 2,000 and one dentist, he made up his mind to stay where providence had located him, and succeed or perish in the effort.

It has since grown to a city of considerable importance with a population of about 25,000, with seventeen or eighteen dentists.

I heard that after making his last and fourth location, he had to borrow more money and took out a life insurance policy to secure payment, and that it took him about seven years from his first start to get out of debt. Since that time I learn he has succeeded fairly well, has a comfortable home, and fairly good living for a small family. Although is he now getting well along in years, I understand he can still be found at his chair, doing work for the grand children of some of his first patients; but not in the old way.

I have been told he keeps well up to date in everything pertaining to his profession.

None can better appreciate the advance and improvements in the profession, and valuable appliances introduced in the last half century, than one who has passed through some of the experiences of this young man.

To the young men I would say, profit by the experience of others, it may save you many a hard knock. First select the town or city you would like to make your home. If you are in debt don't get discouraged if you are not out of debt the first or second year. Be always busy. If you have no patients in the chair, you can always have something doing in the laboratory. People like to patronize the busy man. Stick closely to your office during office hours. Make the last work you do the best word you have ever done. Be a gentleman, always found in good company. If you are not married, marry as soon as you are able to take care of the best girl you ever know. See that she is a good housekeeper. The wife has much to do with making the man a success. Good men make good wives and good wives make good husbands. You may not be able to accumulate a large forture in the practice of your profession, but by close attention to business, you will be able to make a comfortable living for a small family. Collect your bills when due. Work is always more satisfactory when paid for.

